# The Global Burden of Obstructive Sleep Apnea

**DOI:** 10.3390/diagnostics15091088

**Published:** 2025-04-25

**Authors:** Giannicola Iannella, Annalisa Pace, Mario Giuseppe Bellizzi, Giuseppe Magliulo, Antonio Greco, Armando De Virgilio, Enrica Croce, Federico Maria Gioacchini, Massimo Re, Andrea Costantino, Manuele Casale, Antonio Moffa, Jerome R. Lechien, Salvatore Cocuzza, Claudio Vicini, Alberto Caranti, Rosario Marchese Aragona, Mario Lentini, Antonino Maniaci

**Affiliations:** 1 Organi di Senso Department, Sapienza University of Rome, 00161 Rome, Italy; giannicola.iannella@uniroma1.it (G.I.);; 2 Ear, Nose and Throat Unit, Department of Clinical and Molecular Sciences, Polytechnic University of Marche, Via Conca 71, 60020 Ancona, Italy; 3 Department of Otolaryngology—Head and Neck Surgery, AdventHealth Orlando, Orlando, FL 32789, USA; 4 Integrated Therapies in Otolaryngology, Fondazione Policlinico Universitario Campus Bio-Medico, 00128 Rome, Italy; 5 Division of Laryngology and Broncho-Esophagology, Department of Otolaryngology-Head Neck Surgery, EpiCURA Hospital, UMONS Research Institute for Health Sciences and Technology, University of Mons (UMons), 7000 Mons, Belgium; 6 Department of Medical and Surgical Sciences and Advanced Technologies “GF Ingrassia” ENT Section, University of Catania, 95123 Catania, Italy; 7 Department ENT & Audiology, University of Ferrara, 44121 Ferrara, Italy; 8 Otolaryngology Section, Department of Neuroscience, University of Padova, 35100 Padova, Italy; 9 Department of Medicine and Surgery, University of Enna Kore, 94100 Enna, Italyantonino.maniaci@unikore.it (A.M.)

**Keywords:** obstructive sleep apnea, global burden, OSA, sleep, epidemiology

## Abstract

This study reviewed the global prevalence, health and socioeconomic impact, and management approaches of obstructive sleep apnea. The narrative review examined three key dimensions: (1) worldwide OSA prevalence across different regions, accounting for variations in diagnostic standards; (2) OSA’s effects on health outcomes and socioeconomic conditions across diverse populations and healthcare systems; and (3) current global approaches to OSA diagnosis, treatment, and public health management. Despite advances in diagnosis and treatment, a large proportion of OSA cases remain undiagnosed or inadequately managed. The findings show that untreated OSA significantly increases public safety risks, particularly regarding motor vehicle and occupational accidents, while also creating a substantial pool of patients at high risk for systemic complications with severe impacts on overall health. There is a critical need for increased public awareness, universal screening approaches, and integrated care strategies to address this global health challenge and reduce its considerable socioeconomic burden. Our review uniquely addresses global disparities in OSA prevalence, clarifies the health and socioeconomic impacts that remain underexplored in the current literature, and suggests concrete strategies for public health and clinical management improvement worldwide.

## 1. Introduction

### 1.1. Definition and Epidemiology of OSA

Obstructive sleep apnea (OSA) is a serious medical condition, although it is very common, and perhaps one of the most fatal sleep disorders because of repeated cycles of typically above-airway collapse during sleep that result in intermittent hypoxia, sleep fragmentation, and excessive sleepiness during the daytime. It is such a widespread concern that it has been estimated to affect nearly 936 million adults aged between 30 and 69 worldwide [1,2].

### 1.2. Health Impact of OSA, Socioeconomic Impact

The pathophysiologic mechanisms of OSA are numerous-natural and non-natural, which involve muscle reaction, level of arousal, and ventilation control [1,2,3,4,5,6,7,8,9,10,11,12,13,14,15]. The pathogenesis results in several health consequences, such as cardiovascular pathology, metabolic disruptions, cognitive dysfunction, and mood disorders [4,5,6,8,9,10,11]. However, beyond the health effects of individual concern, OSAS is characterized by large socioeconomic costs attributable to direct medical expenditures, treatment, and indirect costs in terms of lost productivity and higher predilection to accidents [15,16].

### 1.3. Current Management and Challenges

Obstructive sleep apnea is an extremely common condition with bad outcome morbidity, but it is yet underdiagnosed because of the evolving diagnostic criteria, low awareness rates, and treatment compliance issues with CPAP therapy [12,14,17,18]. Compounding all these are the inequalities in access to healthcare across systems [19]. Further, public health interventions for OSA are at their infancy stage in most areas, lacking adequate screening programs and policies for implementation, especially in low-resource settings [20,21,22,23,24,25].

This review brings together evidence regarding the prevalence of OSA in all parts of the world and its health effects and socioeconomic implications among various populations and healthcare systems [26,27,28,29]. This narrative review aimed to address three main aspects related to obstructive sleep apnea (OSA): (1) What is the global prevalence of OSA by country, adjusted to the different diagnostic criteria used? (2) What is the effect of OSA on health outcomes and socioeconomic conditions in heterogeneous populations and healthcare systems? (3) What are the contemporary approaches toward the diagnosis, treatment, and public health management of OSA globally? Through this narrative overview and synthesis, we explore the existing literature to highlight gaps in knowledge and suggest key areas of research needed to guide resource allocation and public health action in tackling this global health challenge. Although prior studies have explored OSA epidemiology and impacts separately, this review synthesizes data across diverse populations, diagnostic methods, and healthcare settings, significantly clarifying the global epidemiological landscape and socioeconomic burden of OSA. We intend to bridge knowledge gaps and recognize the direction of future research to resource planning and public health efforts to tackle the worldwide epidemic of OSA [30,31,32,33].

## 2. Materials and Methods

This narrative review summarizes the state of the art on obstructive sleep apnea (OSA), focusing on its epidemiology, health-related burden, economic burden, diagnostic challenges, and public health implications from several perspectives. A comprehensive literature search of the following databases: PubMed, Scopus, Web of Science, and Cochrane Library was conducted. The search included literature published from January 2000 to February 2025. We identified original studies, reviews, guidelines, and expert opinions that were published primarily in the literature. Specifically, inclusion criteria were peer-reviewed articles, systematic reviews, meta-analyses, clinical guidelines, and large cohort studies focusing on the epidemiology, clinical outcomes, economic burden, diagnostic strategies, and management of OSA. No language constriction was considered. On the contrary, exclusion criteria were following study design as case reports, letters to the editor, editorials, conference abstracts, and studies not involving human subjects or lacking relevance to OSA burden.

**Figure 1:**
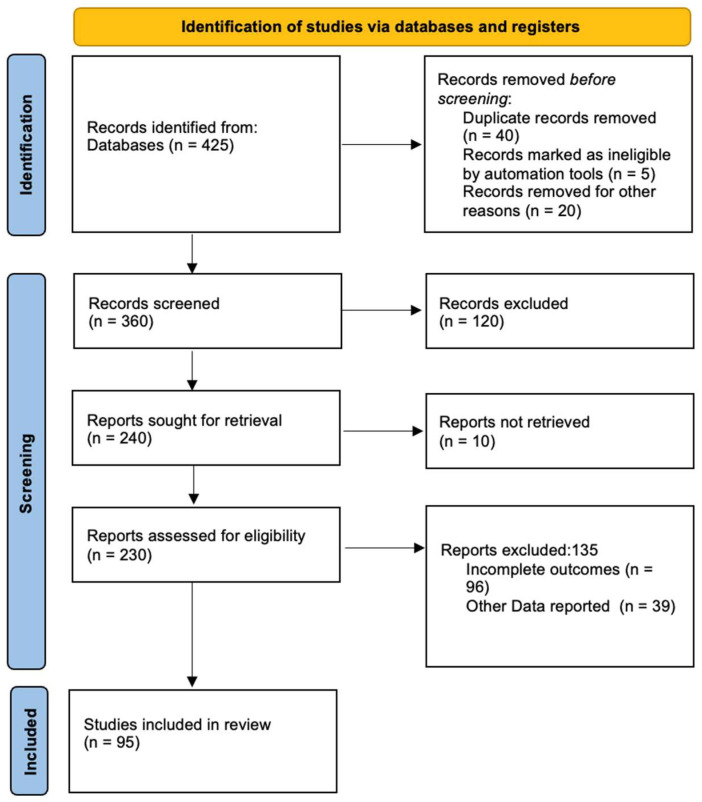
PRISMA 2020 flow diagram of Obstructive Sleep Apnea global burden.

This study has been performed according to PRISMA guidelines (Figure 1)

### 2.1. Literature Selection

We focused on articles deemed relevant to important characteristics of OSA, favoring the literature that added considerable quality to the evidence of OSA consequences in various domains. Sources of evidence included peer-reviewed research articles, clinical practice guidelines, consensus statements, and positional papers in sleep medicine. Particularly emphasized were studies from well-established research teams and clinical centers with demonstrated expertise in sleep-disordered breathing. For epidemiological information, we focused on studying OSA prevalence in various populations and regions of the world. For health effects, we chose the literature describing associations of OSA with different comorbidities, such as cardiovascular, metabolic, neurocognitive, and psychological diseases. The socioeconomic domain was based on health economic analyses, cost of illness studies, and productivity studies. We organized our findings thematically to provide a coherent overview of current knowledge while acknowledging areas of uncertainty and ongoing debate.

### 2.2. Synthesis Approach

Due to the heterogeneity of OSA literature and the wide scope of this review, we used a narrative synthesis approach to collate knowledge from different forms of evidence. In addition, we motivated the study design given the variability in epidemiological methods and diagnostic criteria globally, choosing to integrate diverse findings into a coherent overview, highlighting broader implications for public health and policy. This format enabled us to frame findings in the context of the larger clinical and public health picture and to illuminate links between distinct areas of OSA that may be lost in more narrowly focused analyses. To convey a coherent overview of current knowledge while recognizing areas of uncertainty and ongoing debate, we organized our findings thematically. Instead of systematically covering all specific questions exhaustively like a systematic review, our purpose was more to provide an overview of OSA that encompasses clinical, scientific, and public health perspectives. Where consensus does exist, we focus on time-tested principles; where debate persists, we provide major perspectives from the field. Applying a narrative framework here enabled us to discuss the multifactorial nature of OSA and its related effects through different healthcare settings and patient groups. The review also highlighted existing knowledge gaps and emerging directions to inform future study and clinical practice in this critical domain.

## 3. Results

### 3.1. Global Obstructive Sleep Apnea Prevalence

We initially identified a total of 425 articles through database searches and 15 additional articles via manual reference screening. After the removal of duplicates, 360 articles were screened by title and abstract. Of these, 130 full-text articles were assessed for eligibility, while 95 studies were finally included in the narrative synthesis. Numerous epidemiological studies have assessed the global prevalence of OSA, revealing a complex landscape characterized by notable regional variations and inherent methodological challenges. Recent meta-analyses estimate the prevalence of OSA in adults to range from 9% to 38%, depending on the diagnostic criteria applied [24]. This indicates that a substantial portion of the global population is affected by the condition. However, these figures likely underestimate the true prevalence, particularly in regions with limited access to sleep medicine facilities.

### 3.2. Prevalence Rates by Region

Prevalence rates demonstrate considerable regional disparities, with some of the highest rates observed in North and South America, as well as specific regions of Asia [31]. For instance, a comprehensive study conducted in the United States found that 26% of individuals aged 30 to 70 had at least mild OSA (AHI > 5), while 10% had moderate-to-severe OSA (AHI ≥ 15) [32]. In contrast, estimates of OSA prevalence in European nations are typically lower, ranging from 6% to 17%, although direct comparisons are hindered by methodological differences [33]. Despite generally lower obesity rates than in Western countries, certain Asian populations have exhibited a heightened susceptibility to OSA, with some studies reporting prevalence rates as high as 27.2% in urban Chinese populations [34]. This phenomenon may be partly attributed to craniofacial characteristics that predispose individuals to upper-airway collapse during sleep [35]. Additionally, there remains a notable knowledge gap regarding the global distribution of OSA, as evidenced by the scarcity of data from regions such as Africa and parts of South America [36].

### 3.3. Demographic Factors and Risk Factors

The prevalence of OSA is significantly influenced by demographic factors, with age, gender, and ethnicity emerging as key variables. Age is a critical factor in the occurrence of OSA; studies suggest that up to 90% of men and 78% of women over the age of 70 may exhibit at least mild OSA [37]. Most studies indicate a male-to-female ratio of 2:1 to 3:1 in the general population. However, this difference narrows in older age groups and may be influenced by the underdiagnosis of OSA in women [38]. Gender disparities in OSA prevalence are well documented. Furthermore, the prevalence of OSA is significantly influenced by socioeconomic status (SES), although the nature of this association is complex and may vary across different populations [39]. Some research indicates a negative correlation between OSA prevalence and SES, potentially mediated by factors such as obesity, smoking, and healthcare access [40]. However, the lack of consistency in this relationship across studies underscores the need for further investigation into the socioeconomic determinants of OSA.

### 3.4. Clinical Aspects and Diagnostic Criteria and Methods

Accurately determining the global prevalence of OSA remains challenging for several reasons. First, significant variability in prevalence estimates arises from differences in diagnostic criteria over time [41] (Table 1).

Second, underdiagnosis is a concern, as polysomnography—the gold standard for diagnosing OSA—is resource-intensive and unavailable in many regions [14]. The increasing availability of home sleep apnea testing (HSAT) devices has facilitated diagnosis; however, due to variations in sensitivity and specificity when compared to type II or III polysomnography, prevalence estimates may vary [42]. Lastly, many prevalence studies rely on questionnaire-based screening tools, which, while useful for large-scale epidemiological surveys, may not fully capture the spectrum of OSA severity [43]. Despite these challenges, recent efforts to compile global prevalence statistics have provided valuable insights into the widespread nature of OSA. A key study by Benjafield et al., (2019) estimated that 425 million adults worldwide suffer from moderate-to-severe OSA (AHI ≥ 15), while 936 million individuals aged 30–69 have mild to severe OSA (AHI ≥ 5) [2]. These figures highlight the global significance of OSA as a public health issue and underscore the urgent need for improved prevention, diagnosis, and treatment strategies across diverse patient populations and healthcare systems (Table 2).

### 3.5. The Effects of Obstructive Sleep Apnea on Health and Pathophysiology

OSA exerts significant systemic repercussions that profoundly impact general health and quality of life beyond the immediate symptoms of sleep disruption. Multiple organ systems are affected by a cascade of pathophysiological mechanisms initiated by recurrent episodes of intermittent hypoxia, intrathoracic pressure swings, and sleep fragmentation [44]. A major and well-documented consequence of OSA is its effect on cardiovascular health. Both cross-sectional and longitudinal studies consistently demonstrate a strong association between OSA and hypertension, with evidence suggesting a dose–response relationship between the severity of OSA and increased blood pressure [45]. The landmark Wisconsin Sleep Cohort study highlighted that individuals with an apneas–hypopneas index (AHI) ≥ 15 had a threefold higher risk of developing hypertension over a four-year follow-up period, independent of other established risk factors [46]. Additionally, research suggests that up to 83% of patients with resistant hypertension remain undiagnosed for OSA, indicating that OSA may play a significant role in the pathophysiology of resistant hypertension [47]. In addition to hypertension, OSA is associated with an increased risk of other cardiovascular conditions. Cohort studies adjusted for traditional cardiovascular risk factors have found that individuals with severe OSA exhibit a significantly higher prevalence of coronary artery disease, with hazard ratios ranging from 1.5 to 4.5 [48]. Moreover, OSA has been linked to an elevated risk of heart failure; one study reported a 140% higher risk of heart failure in men with severe OSA compared to those without sleep disturbances [49]. OSA has also been associated with an increased incidence of arrhythmias, particularly atrial fibrillation, stroke, and sudden cardiac death [50,51].

The metabolic implications of OSA are similarly concerning, with robust evidence connecting the condition to insulin resistance, glucose intolerance, and type 2 diabetes. A meta-analysis of prospective cohort studies, adjusting for BMI and other confounding factors, revealed that moderate-to-severe OSA was associated with a 63% increased risk of developing type 2 diabetes [52]. Furthermore, intermittent hypoxia, a hallmark of OSA, has been suggested to exacerbate hepatic steatosis and fibrosis, which may contribute to the onset and progression of non-alcoholic fatty liver disease (NAFLD) [53].

OSA also has significant neurocognitive effects, impairing attention, memory, and executive function. The intermittent hypoxia and chronic sleep fragmentation characteristic of OSA have been linked to significant deficits on neuropsychological tests, with the most pronounced effects observed in attention/vigilance and executive function domains, as reported in a meta-analysis of 42 studies [54]. These cognitive deficits raise substantial safety concerns and can detract from daily productivity. Untreated OSA patients are at an increased risk of motor vehicle accidents, with one meta-analysis indicating that their risk is 2.4 times higher compared to controls [55].

There has been increasing attention given to the relationship between OSA and mental health disorders. The prevalence of depressive symptoms among individuals with OSA has been reported to range from 7% to 63%, depending on the population and diagnostic criteria used [56]. A reciprocal relationship between OSA and depression has been suggested, where depression may increase the risk of OSA through mechanisms such as weight gain and reduced physical activity, while OSA may exacerbate depressive symptoms through disrupted sleep and altered neurotransmitter function [57]. Anxiety disorders are also more prevalent in individuals with OSA, with one study showing that 53.9% of newly diagnosed OSA patients had anxiety disorders, compared to 16.7% in controls [58].

The overall quality of life for individuals with OSA can be profoundly impacted by the condition. Patients frequently experience excessive daytime sleepiness, fatigue, and diminished energy, all of which can severely impair social interactions and workplace productivity [59]. Furthermore, OSA has been associated with sexual dysfunction, particularly erectile dysfunction in men, further compromising the quality of life [60]. Importantly, individuals with untreated severe OSA face a significantly higher risk of mortality. The seminal study by Young et al. demonstrated that severe OSA (AHI > 30) was associated with a 3.8-fold increase in all-cause mortality over an 18-year follow-up period [61]. These findings have been corroborated by subsequent research, with one meta-analysis revealing a pooled hazard ratio of 1.54 for all-cause mortality in individuals with OSA compared to controls [62].

Encouragingly, many of the detrimental health effects associated with OSA may be mitigated with appropriate treatment, particularly with CPAP therapy. Studies have shown that CPAP therapy improves a range of health outcomes, including quality of life, insulin sensitivity, blood pressure regulation, and cognitive performance [63,64]. However, further large-scale, long-term, randomized controlled trials are required to definitively establish the long-term cardiovascular benefits of OSA treatment [65].

### 3.6. The Socioeconomic Impact of Obstructive Sleep Apnea

OSA imposes substantial socioeconomic costs, encompassing direct medical expenses, indirect costs related to accidents and reduced productivity, and broader societal implications. As the global prevalence of OSA continues to rise, understanding and quantifying these economic impacts has become increasingly critical for healthcare policy development and resource allocation.

The direct medical expenses associated with OSA are considerable, arising from various diagnostic and treatment-related sources. Key diagnostic procedures, including polysomnography and home sleep apnea testing, contribute significantly to these costs [14]. A study conducted in the United States in 2015 estimated that the annual cost of diagnosing OSA amounted to nearly USD 2.4 billion [66]. The financial burden is further exacerbated by treatment expenditures, particularly those related to CPAP therapy. The annual cost of CPAP therapy in the United States has been estimated at USD 3.4 billion, with projections indicating that this amount could increase to USD 11.1 billion by 2030 due to rising OSA prevalence [67] (Table 3).

**Figure 2:**
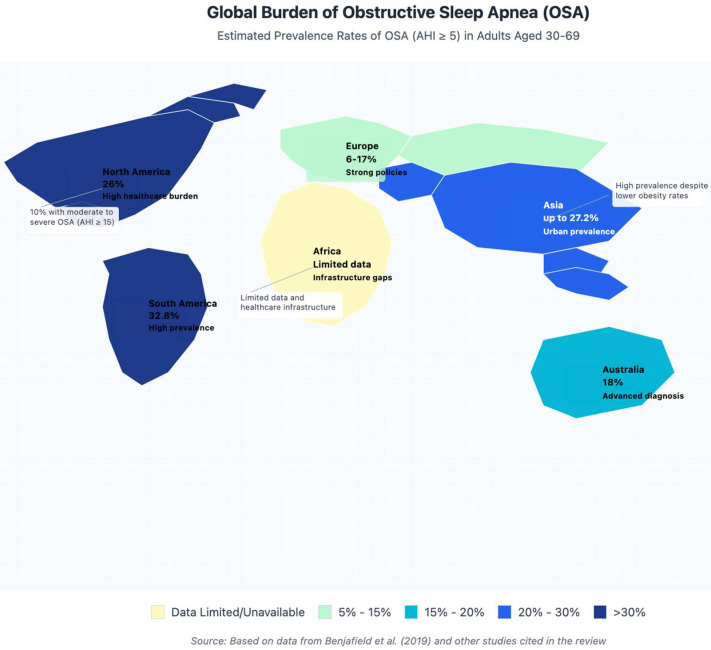
Estimated prevalence of OSAS worldwide, Benjafield et al., 2019 [2].

In addition, individuals with OSA experience higher healthcare utilization across various medical specialties. Research has demonstrated that untreated OSA patients have significantly higher rates of hospital admissions, emergency room visits, and medication use compared to those without OSA [68]. One large retrospective cohort study found that healthcare costs in the year preceding an OSA diagnosis were nearly twice as high for patients compared to matched controls [69]. Even after diagnosis, OSA patients continue to exhibit higher healthcare consumption and associated costs compared to the general population [70] (Figure 2).

The indirect costs of OSA, while more challenging to quantify, are thought to far exceed direct medical expenses. The financial impact of lost productivity due to excessive daytime sleepiness and cognitive impairment is substantial. A study conducted in the United States projected that the combined annual cost of presenteeism, absenteeism, and lost productivity due to OSA was USD 86.9 billion in 2015 [67]. This amount underscores the significant economic impact of OSA beyond the healthcare sector, as it exceeds the direct medical costs associated with the disorder.

Another important indirect cost of OSA is occupational hazards. Untreated OSA has been linked to an increased risk of workplace accidents, particularly in safety-sensitive occupations such as manufacturing and commercial driving [10]. A meta-analysis reported that employees with OSA were nearly twice as likely to experience work-related accidents compared to those without OSA [71]. These incidents have economic ramifications beyond the immediate costs of property damage and injuries, including lost productivity, workers’ compensation claims, and potential legal penalties.

The association between OSA-related fatigue and motor vehicle accidents (MVAs) presents a significant public health and economic concern. Multiple studies have consistently shown that individuals with untreated OSA are 2–3 times more likely to be involved in MVAs compared to those without the condition [72]. A study in the United States estimated that OSA-related motor vehicle accidents result in USD 15.9 billion in collision expenses and 1400 fatalities annually [73]. The economic consequences of these accidents are profound, encompassing not only direct costs but also broader societal impacts.

OSA also affects resource allocation within healthcare systems, potentially exacerbating health inequalities. The high prevalence of undiagnosed OSA places considerable strain on primary care and specialized services, leading to delays in the diagnosis and treatment of other conditions and longer wait times for care [74]. Moreover, the complex interplay between OSA and comorbidities, such as type 2 diabetes and cardiovascular disease, suggests that effective management of OSA could result in significant financial savings in the treatment of these related conditions [75].

From a societal perspective, OSA imposes a significant financial burden, particularly in the context of family dynamics. Partners of individuals with OSA often report disrupted sleep and a reduced quality of life, contributing to additional productivity losses and higher medical expenses [76]. Furthermore, the increased risk of occupational and vehicular accidents associated with OSA affects public safety and insurance systems [77].

Despite the substantial economic burden of OSA, studies have consistently demonstrated the cost-effectiveness of diagnosing and treating moderate-to-severe cases. CPAP therapy, in particular, has been shown to yield favorable cost-effectiveness ratios across various healthcare systems and economic settings [78]. These findings suggest that increased investment in the diagnosis and treatment of OSA could result in substantial financial benefits for both individuals and society.

However, significant barriers remain to realizing these potential financial gains. Research indicates that up to 80% of individuals with moderate-to-severe OSA remain undiagnosed, highlighting the critical issue of underdiagnosis [79]. Addressing this gap will require increasing awareness among both the public and healthcare providers, as well as the development of more affordable and accessible diagnostic methods. Furthermore, strategies to improve long-term adherence to CPAP therapy are essential, as insufficient compliance presents a major obstacle to fully realizing the economic benefits of OSA treatment [80].

In conclusion, the socioeconomic effects of OSA are extensive and multifaceted, extending well beyond the direct medical costs of diagnosis and treatment. The significant indirect costs associated with workplace accidents, reduced productivity, and broader societal impacts underscore the importance of addressing OSA as a serious public health and financial issue. Although challenges remain, the proven cost-effectiveness of OSA treatment suggests that increased funding for diagnosis and management could result in substantial financial gains for both individuals and society as a whole.

### 3.7. Diagnosis and Treatment of Obstructive Sleep Apnea: An Evolving Landscape

The diagnosis and treatment of OSA are characterized by regional variability, technological advancements, and persistent challenges related to accessibility and treatment adherence. Understanding these factors is crucial for addressing the global burden of OSA effectively.

Recent developments in OSA diagnostic techniques reflect the need to balance diagnostic accuracy with cost-effectiveness and accessibility. Polysomnography (PSG), conducted in a sleep laboratory, remains the gold standard for diagnosing OSA, providing comprehensive data on respiratory events, sleep architecture, and associated physiological factors [14]. However, due to the high cost and limited availability of in-laboratory testing, home sleep apnea testing (HSAT) has emerged as a more accessible alternative. While HSAT typically measures fewer physiological parameters than PSG, it has proven to be a cost-effective diagnostic tool for select patient populations [81]. The American Academy of Sleep Medicine (AASM) endorses HSAT as an acceptable diagnostic test for individuals without significant comorbidities who exhibit signs and symptoms of OSA [14]. However, regional and healthcare system-specific variations in HSAT implementation persist. In countries like the United States and Canada, HSAT is frequently employed as the first-line diagnostic test for suspected OSA [82]. In contrast, many European countries and regions in Asia continue to rely predominantly on in-laboratory PSG due to concerns about the potential for underdiagnosing certain patient groups with HSAT [83].

Diagnostic criteria for OSA have evolved over time, influencing both prevalence estimates and treatment recommendations. For example, the AASM’s 2012 guidelines recommended reducing the oxygen desaturation threshold for assessing hypopneas from 4% to 3%, resulting in higher estimates of OSA prevalence and severity [84]. This shift has implications for treatment strategies and resource allocation, particularly in healthcare systems with limited resources for OSA management.

Treatment options for OSA have expanded, but CPAP therapy remains the cornerstone of treatment for moderate-to-severe cases. CPAP has been well documented to improve subjective daytime sleepiness and reduce the apnea–hypopnea index (AHI) [63]. However, large randomized controlled trials have produced mixed results regarding the long-term impact of CPAP on cardiovascular outcomes, and this remains an area of ongoing debate [64,65]. Alternative therapies for OSA are increasingly utilized, particularly for patients who cannot tolerate or adhere to CPAP therapy. Oral appliances have demonstrated efficacy in treating mild to moderate OSA, and some severe OSA patients who are intolerant to CPAP have also benefited from these devices [85]. These appliances function by advancing the mandible to increase the upper airway’s patency. Surgical interventions can be effective for certain patients, but they are generally reserved for highly selected individuals [86], with procedures ranging from soft tissue surgeries to maxillomandibular advancement. Emerging treatments, such as hypoglossal nerve stimulation, have shown promise in specific patient populations. For individuals with moderate-to-severe OSA who cannot use CPAP, hypoglossal nerve stimulation has been associated with improved quality of life and reductions in AHI [87]. However, the high cost of this treatment and the lack of long-term data limit its widespread adoption.

Barriers to diagnosis and treatment exist both in high-income and low- and middle-income countries (LMICs). In high-income nations, challenges include delays in initiating treatment and extended waiting times for sleep testing [88]. In LMICs, more substantial barriers exist, including limited access to diagnostic tools, treatment resources, and sleep medicine specialists [89]. A survey of 36 nations revealed significant disparities in the availability of sleep laboratories, with some countries reporting as few as 0.1 sleep laboratories per 100,000 people, while others had over 0.9 sleep laboratories per 100,000 people [2].

Economic evaluations have consistently demonstrated the cost-effectiveness of diagnosing and treating moderate-to-severe OSA. CPAP therapy has been linked to favorable cost-effectiveness ratios across various healthcare systems and economic contexts, accounting for both direct healthcare expenses and indirect costs such as accidents and lost productivity [78]. However, the potential economic benefits of OSA treatment remain underutilized due to poor adherence to CPAP therapy. Studies indicate that between 29% and 83% of patients do not comply with CPAP usage guidelines, which define adherence as using the device for at least 4 h per night on 70% of nights [80]. Current research and clinical practice are focused on improving CPAP adherence through educational interventions, technological advancements, and individualized treatment strategies [90].

Technological advancements and the push for personalized medicine are expected to significantly influence the future of OSA diagnosis and treatment. Machine learning algorithms, utilizing data from home sleep tests, have shown promise in enhancing diagnostic accuracy and predicting treatment outcomes [91]. Furthermore, advancements in CPAP technology, such as auto-titrating devices and remote monitoring capabilities, offer the potential to improve patient compliance and treatment effectiveness [92]. The application of phenotyping techniques, which account for the variability in OSA presentations, is anticipated to lead to more tailored and effective treatment approaches [93].

### 3.8. Strategies and Policies in Public Health for Obstructive Sleep Apnea

To address the prevention, early detection, and management of OSA, comprehensive public health strategies and policies are required. Despite the significant health and economic burden imposed by OSA, it remains insufficiently recognized as a public health issue in many regions. Effective public health initiatives are essential for mitigating the impact of OSA on individual health, healthcare systems, and society at large.

Public health efforts for OSA primarily focus on awareness and education campaigns. Despite the high prevalence of OSA, public awareness remains low in many parts of the world [94]. For instance, a survey conducted across five countries—Australia, Japan, Korea, Singapore, and Thailand—found that only 9% of respondents had heard of OSA, with significant variation across countries [95]. This lack of awareness contributes to delays in diagnosis and treatment. Educational campaigns have shown promise in raising awareness and encouraging individuals to seek medical attention. For example, a community-based education initiative in the United States led to a notable increase in OSA diagnoses and the implementation of sleep studies [96].

Healthcare provider education is equally critical, as primary care physicians and non-sleep specialists often lack adequate knowledge about OSA diagnosis and management. Research suggests that integrating OSA education into medical school curricula and offering continuing medical education (CME) for practicing physicians can significantly improve the identification and management of OSA [97]. Despite some global initiatives, there have been limited efforts to incorporate sleep medicine into primary care education and practice [98].

Screening for OSA has gained traction as a potential strategy for enhancing early detection and intervention. Several screening tools, such as the Berlin Questionnaire and the STOP-Bang questionnaire, have been developed and shown to be effective in identifying individuals at high risk for OSA [99]. These tools have demonstrated good sensitivity in detecting moderate-to-severe OSA and are easily implementable in primary care settings. Some healthcare systems have introduced systematic screening programs targeting high-risk populations, such as individuals with obesity, hypertension, or type 2 diabetes [100].

### 3.9. Socioeconomic Burden, Disease Prevention, and Control Initiatives

Debate continues regarding the most cost-effective method for population-wide OSA screening, with concerns about overdiagnosis and resource utilization [101].

Workplace screening and intervention programs for OSA have emerged as a significant public health strategy, particularly in occupations where worker safety is a priority. The transportation sector has been at the forefront of such initiatives, with some countries mandating OSA screening for commercial drivers [102]. For instance, in the European Union, OSA screening is required as part of the medical evaluation for obtaining a commercial driver’s license [103]. Similar programs are being considered for other high-risk professions, including pilots and railroad workers [104].

Professional organizations and health bodies have proposed policy recommendations to address the public health impact of OSA. The American Academy of Sleep Medicine (AASM) has advocated for legislation that increases access to OSA diagnosis and treatment, including enhanced insurance coverage for OSA services and the recognition of OSA as a chronic disease [105]. Similarly, the European Respiratory Society (ERS) has highlighted the need for improved access to care, better training for healthcare professionals, and heightened public awareness [106].

One potential avenue for optimizing existing public health infrastructure is integrating OSA management into broader chronic disease prevention and control initiatives. Given the substantial comorbidities between OSA and conditions such as obesity, cardiovascular disease, and diabetes, there is an opportunity to incorporate OSA screening and care into established chronic disease management programs [107]. Although some healthcare systems have begun implementing such integrated approaches, their widespread adoption remains limited [6].

International collaboration and initiatives are crucial to reducing the global prevalence of OSA. The World Sleep Society (formerly the World Association of Sleep Medicine) has played a pivotal role in raising awareness about sleep disorders, including OSA, through its World Sleep Day campaign and educational programs [108]. Furthermore, the World Health Organization (WHO) recognized the importance of sleep health, including OSA management, in its Global Action Plan for the Prevention and Control of Noncommunicable Diseases [109]. However, many global health agendas and frameworks still fail to specifically address sleep disorders like OSA.

Research focused on improving the understanding of OSA’s global epidemiology and impact is essential for informing public health policies. The Sleep and Health in Women project, a large-scale global partnership, has provided valuable data on OSA prevalence and risk factors in women across multiple countries [110]. Additionally, the European Sleep Apnea Database (ESADA) has contributed to shaping clinical practice guidelines and enabling comparative effectiveness research on OSA management [111].

Addressing OSA in low- and middle-income countries (LMICs) presents unique challenges due to limited access to diagnostic resources, treatment options, and sleep medicine specialists. Initiatives like the “Sleep Apnea Global Initiative” aim to develop and validate simplified diagnostic algorithms and treatment pathways suitable for resource-limited settings [112,113,114,115,116,117,118]. However, significant barriers remain, including the need for sustained commitment to managing OSA as a chronic condition, fragmented healthcare systems, and conflicting health priorities in many countries. Furthermore, the COVID-19 pandemic has underscored the vulnerabilities of sleep health management, highlighting both the opportunities and risks associated with disruptions in sleep services [113,114,115,116,117,118,119,120,121].

## 4. Discussion

Several key considerations that should be examined further arise from the synthesis of current evidence regarding OSA. While the existing literature often assumes uniform diagnostic criteria and epidemiological approaches, our synthesis clearly highlights that substantial variation in prevalence data across regions stems from significant methodological inconsistencies, demographic diversity, and unequal healthcare access.

Consequently, prior estimates may significantly underestimate the true global burden of OSA.

Our comprehensive review advances current scholarship by explicitly synthesizing global epidemiological variations, health impacts, socioeconomic burdens, and diagnostic challenges into a coherent framework. This multidimensional perspective significantly clarifies the complex interactions between epidemiological determinants and clinical outcomes that have previously been examined only in isolation. The substantial variability in prevalence by region and population suggests a complex interplay of genetic, environmental, and sociocultural factors [24,31,34,122,123,124,125,126,127]. Although baseline prevalence figures have been established through epidemiological studies, understanding how these determinants intersect with each other is key to addressing targeted interventions and increasing public awareness.

Contrary to the assumption in previous research that OSA prevalence primarily correlates with obesity prevalence, our analysis emphasizes the critical role of demographic and craniofacial variations, particularly in Asian populations, indicating the necessity of more nuanced and culturally sensitive diagnostic and management strategies.

Of grave concern, however, is the general therapeutic lag in OSA management as identified by the current data, whereby ideas on best practices are not adopted into the clinic. In spite of strong evidence for early intervention, the number of undiagnosed cases indicates a systematic barrier to implementing early detection and intervention, suggesting that a universal screening protocol should be proposed [79,80,128,129,130,131,132,133,134]. These barriers are likely multifactorial and involve healthcare system-level issues, provider education gaps, and patient-level factors that should be incorporated within policy framework development. The societal costs of untreated OSA provide a powerful moral imperative for systemic change [135,136,137,138]. In addition to individual health effects, specified effects on workplace safety, transportation accidents, and healthcare utilization are also causing economic concern with current OSA management pathways, which may prove to be economically unfeasible in the long term [71,72,73]. This economic burden is especially acute in resource-poor settings in which diagnostic and therapeutic infrastructure may not be sufficient [88,89].

While promising, OSA devices available both for diagnosis and treatment also pose new challenges related to standardization and accessibility, which need to be addressed as explored in [91,92]. The widespread availability of home sleep testing and non-traditional treatments has expanded the range of options for care but has also created the potential for disparities in quality and outcomes. Thus, these trends warrant a nuanced exploration for reconciling innovation and health equity in service delivery [93,94].

Our review explicitly addresses several previously underexplored knowledge gaps, notably global disparities in diagnostic infrastructure, significant socioeconomic impacts related to productivity and safety risks, and systemic barriers to public health intervention. Highlighting these gaps is essential to prioritizing areas for immediate policy action and resource allocation.

An important shift in perspective is the increasing recognition in the global health community regarding the importance of OSA, as demonstrated by its incorporation into the WHO’s noncommunicable disease framework [109].

Given the significant variability in diagnostic practices and the widespread socioeconomic burden of obstructive sleep apnea, our findings underscore the urgent need for standardized yet flexible global diagnostic frameworks. Improving accessibility to cost-effective diagnostic tools and implementing targeted public health policies are essential steps toward reducing disparities in care. Despite growing recognition of OSA as a global health issue, reflected in initiatives by the WHO and other organizations, this awareness has yet to translate into coordinated, actionable strategies [139,140]. Current efforts remain fragmented and insufficiently integrated across health systems. To effectively address the global impact of OSA, stronger international cooperation, shared guidelines, and unified health policies are required.

### Future Direction and Challenges

Rapid advances in machine learning (ML) and artificial intelligence (AI) are transforming the diagnosis and treatment of OSA. Recent validation experiments [141,142] featuring state-of-the-art ML algorithms incorporating complex multimodal data (ECG, oximetry, and acoustic) herald a world with potential for automatic diagnosis. Novel deep learning architectures [143] are advancing OSA severity classification and clinical outcome prognostication, but barriers to real-world implementation persist. Seventh, next-generation smart wearables [144] are an emerging paradigm in sleep monitoring. Ground-breaking investigations revealed several OSA phenotypes, each with a unique cardiovascular risk signature [145,146], heralding a new era in personalized medicine. Although this phenotyping method holds promise for individualized CPAP protocols [147], considerable hurdles remain with respect to algorithmic fairness and equitable healthcare access [148], which call for creative solutions for underserved individuals. Cutting-edge integrated OSA care has opened up a new paradigm in chronic care of modality-based treatment (M-MT) [149]. Innovative multi-specialty collaborative models [150] are changing the traditional treatment paradigms. Innovative therapeutic options continue to be examined in the emerging data showcasing cardiology–sleep medicine collaborations [151,152].

Advanced diagnostic algorithms [153] are revolutionizing risk prediction, although implementation challenges remain. Another exciting example of a radical digital solution to CPAP therapy [154] is “adaptive” therapy systems and AI-based systems that coach and support the patient. Novel research [154] delineates complex bidirectional relationships between OSA and several neurological conditions, with implications for new therapeutic targets.

## 5. Conclusions

OSA represents a major and growing global health concern with far-reaching clinical and socioeconomic consequences. Although its recognition has increased in recent years, the condition remains substantially underdiagnosed and undertreated, especially in low-resource settings. The burden of OSA extends beyond individual health, contributing to increased morbidity and mortality, strain on healthcare systems, and significant indirect costs associated with reduced productivity and impaired quality of life. Timely diagnosis and appropriate treatment, particularly with continuous positive airway pressure (CPAP) therapy, have proven effective in improving clinical outcomes and reducing long-term costs. To address these challenges, it is essential to implement standardized diagnostic frameworks, expand access to affordable diagnostics and therapies, and raise awareness among healthcare providers and the public. Achieving meaningful progress will require coordinated action at national and international levels, with a shared commitment to reducing the burden of OSA and promoting health equity.

## Figures and Tables

**Table 1:** Summary of selected studies reporting OSA prevalence across different regions, highlighting the variation based on diagnostic approaches and population characteristics.

Region/Country	Study (Author, Year)	Sample Size	Diagnostic Criteria (AHI)	Prevalence (%)	Notes/Key Findings
United States	Peppard et al., 2013 [32]	1520	AHI ≥ 5/≥15	26%/10%	Higher in males, aged 30–70
Europe (multiple)	Franklin et al., 2015 [12]	Varies	AHI ≥ 5/≥15	6–17%	Lower than North America
China (urban)	Ip et al., 2001 [34]	153	AHI ≥ 5	27.2%	Despite lower obesity rates
Nigeria	Adewole et al., 2009 [36]	248	Questionnaire + AHI est.	~12%	Limited diagnostic access
Global (meta-analysis)	Benjafield et al., 2019 [2]	—	AHI ≥ 5/≥15	936 M/425 M	Global burden estimate

**Table 2:** Global burden of obstructive sleep apnea.

Study	Design	Sample Size	Population	Location	Study Period	Primary Outcome	Key Findings	Inclusion Criteria	Exclusion Criteria	Limitations	GRADE Score
Peppard et al., 2013 [32]	Prospective cohort	1520	Adults aged 30–70 in the U.S.	USA	1988–2011	OSA prevalence	26% have mild OSA (AHI > 5), 10% have moderate–severe OSA (AHI ≥ 15)	Adults aged 30–70 from Wisconsin Sleep Cohort	Not specified	Single geographic area, potential selection bias	Moderate
Young et al., 2008 [43]	Prospective cohort	1522	Wisconsin Sleep Cohort	USA	1989–2008	All-cause mortality	Severe OSA associated with 3.8-fold increased mortality risk	Participants of Wisconsin Sleep Cohort	Those lost to follow-up	Single geographic area, potential confounding factors	Moderate
Marin et al., 2005 [44]	Observational	1387	Men with OSA	Spain	1992–1999	Cardiovascular events	Untreated severe OSA increased cardiovascular risk	Men referred for suspected sleep breathing disorders	Central sleep apnea, previous CVD	Male-only population, potential selection bias	Low
McEvoy et al., 2016 [45]	Randomized controlled trial	2717	Adults with moderate–severe OSA	Australia, China, New Zealand, Spain	2008–2013	Cardiovascular events	CPAP did not prevent cardiovascular events	Adults 45–75 years with moderate–severe OSA and coronary or cerebrovascular disease	Severe sleepiness, severe hypoxemia	Limited generalizability to all OSA patients	High
Gottlieb et al., 2010 [46]	Prospective cohort	4422	Sleep Heart Health Study participants	USA	1995–2006	Incident heart failure	OSA associated with increased heart failure risk in men	Adults ≥ 40 years without heart failure at baseline	Central sleep apnea	Self-reported heart failure outcomes	Moderate
Albarrak et al., 2005 [47]	Retrospective cohort	342	Men using CPAP for OSA	Canada	1994–1999	Healthcare utilization	Increased utilization in 5 years before OSA diagnosis	Men diagnosed with OSA and prescribed CPAP	Incomplete data, non-compliance with CPAP	Male-only, retrospective design	Low
Strollo et al., 2014 [48]	Prospective cohort	126	Adults with moderate–severe OSA	Multiple centers in the USA and Europe	Not specified	OSA severity (AHI)	Upper-airway stimulation improved OSA outcomes	Adults with moderate–severe OSA intolerant to CPAP	Central sleep apnea, obesity	Strict inclusion criteria, potential selection bias	Moderate
Ip et al., 2001 [34]	Cross-sectional	153	Middle-aged Chinese men in Hong Kong	Hong Kong	Not specified	OSA prevalence	High OSA prevalence in Asian populations	Chinese men aged 30–60 years	Known sleep disorders, major health problems	Male-only, single ethnic group	Low
Pedrosa et al., 2011 [49]	Cross-sectional	125	Patients with resistant hypertension	Brazil	Not specified	OSA prevalence	High prevalence of undiagnosed OSA in resistant hypertension	Adults with resistant hypertension	Secondary hypertension, kidney disease	Referral population, potential selection bias	Low
Chen et al., 2015 [39]	Cross-sectional	6174	Multi-Ethnic Study of Atherosclerosis participants	Multiple sites in USA	2010–2013	Sleep disturbances	Racial/ethnic differences in sleep disturbances including OSA	MESA participants aged 45–84 years	Clinical cardiovascular disease	Cross-sectional design, self-reported measures	Moderate
Tarasiuk et al., 2008 [50]	Retrospective cohort	289	Middle-aged and older adults with OSA	Israel	2001–2003	Healthcare utilization	Increased morbidity and healthcare use in OSA patients	Adults diagnosed with OSA	Central sleep apnea, incomplete data	Retrospective design, potential confounding factors	Low
Jennum & Kjellberg, 2011 [51]	Controlled national study	19,438	Danish population	Denmark	1998–2006	Socioeconomic consequences	OSA associated with increased health-related costs	All Danish citizens diagnosed with sleep disorders	Not specified	Reliance on national registers, potential misclassification	Moderate
Rezaeitalab et al., 2014 [52]	Cross-sectional	178	Newly diagnosed OSA patients	Iran	Not specified	Anxiety disorders	High prevalence of anxiety disorders in OSA patients	Adults newly diagnosed with OSA	Previous psychiatric disorders, other sleep disorders	Cross-sectional design, single-center study	Low
Sassani et al., 2004 [53]	Retrospective analysis	N/A	U.S. population	USA	2000	Motor vehicle collisions	Estimated high costs due to OSA-related collisions	N/A (population-based analysis)	N/A	Reliance on estimates, potential overestimation	Very low
Franklin et al., 2013 [54]	Cross-sectional	400	Swedish women	Sweden	2000–2004	OSA prevalence	High prevalence of OSA in females	Women aged 20–70 years	Pregnancy, hormonal therapy	Limited geographic area, potential selection bias	Low
Hedner et al., 2011 [55]	Cross-sectional	5103	European Sleep Apnoea Database	Multiple centers in Europe	2007–2009	OSA characteristics	Characteristics of OSA patients across Europe	Adults referred for sleep studies	Not specified	Referral population, potential selection bias	Moderate
Taranto-Montemurro et al., 2019 [56]	Randomized controlled trial	20	Adults with OSA	USA	Not specified	OSA severity (AHI)	Combination therapy reduced OSA severity	Adults with OSA (AHI 20–50)	Severe obesity, other sleep disorders	Small sample size, short-term follow-up	High
Marcus et al., 2013 [57]	Randomized controlled trial	464	Children with OSA	USA	2007–2011	OSA symptoms and quality of life	Adenotonsillectomy improved outcomes in childhood OSA	Children 5–9 years with OSA	Severe OSA, obesity, craniofacial abnormalities	Limited age range, exclusion of severe cases	High
Zanobetti et al., 2010 [58]	Cross-sectional	6441	Adults from seven U.S. urban areas	USA	1995–1998	Sleep-disordered breathing	Association between air pollution and sleep-disordered breathing	Adults from Sleep Heart Health Study	Missing pollution or sleep data	Cross-sectional design, potential confounding factors	Low

**Table 3:** Overview of the major economic consequences linked to untreated OSA, emphasizing the magnitude of both direct medical expenses and indirect societal costs.

Economic Impact Category	Study/Source	Country	Estimated Cost/Effect	Notes
Direct Diagnosis Costs	Watson et al., 2016 [16]	USA	USD 2.4B annually	Polysomnography, home testing
CPAP Therapy Costs	Frost & Sullivan, 2016 [67]	USA	USD 3.4B annually (projected USD 11.1B by 2030)	Growing due to higher prevalence
Healthcare Utilization	Albarrak et al., 2005 [47]	Canada	↑ hospitalization and ER visits	5-year pre- vs. post-diagnosis analysis
Lost Productivity	Frost & Sullivan, 2016 [67]	USA	USD 86.9B annually	Includes presenteeism, absenteeism
Motor Vehicle Accidents	Sassani et al., 2004 [53]	USA	USD 15.9B + 1400 fatalities/year	Attributable to untreated OSA
Workplace Accidents	Garbarino et al., 2016 [10]	Global (meta)	2× risk of workplace injury	Safety-sensitive occupations

↑, Increase in hospitalization.

## Data Availability

Data are unavailable due to privacy or ethical restrictions.
